# Dexmedetomidine expands monocytic myeloid-derived suppressor cells and promotes tumour metastasis after lung cancer surgery

**DOI:** 10.1186/s12967-018-1727-9

**Published:** 2018-12-11

**Authors:** Xiaosan Su, Yaodong Fan, Liu Yang, Jie Huang, Fei Qiao, Yu Fang, Jun Wang

**Affiliations:** 1 Biomedical Research Center, Affiliated Calmette Hospital of Kunming Medical University, 504 Qing Nian Road, Kunming, 650011 Yunnan People’s Republic of China; 2 Department of Neurosurgery, Third Affiliated Hospital of Kunming Medical University (Yunnan Cancer Hospital), 519 Kun Zhou Road, Kunming, 650118 Yunnan People’s Republic of China; 3 Department of Anesthesiology, First Affiliated Hospital of Kunming Medical University, 295 Xi Chang Road, Kunming, 650032 Yunnan People’s Republic of China

**Keywords:** Dexmedetomidine, Myeloid-derived suppressor cells, Lung cancer, Metastasis, Perioperative care

## Abstract

**Background:**

Dexmedetomidine (DEX) has been reported to promote tumour metastases. However the underlying mechanisms remain unclear. The purpose of this study was to investigate whether DEX promotes tumour metastasis by inducing myeloid-derived suppressor cells (MDSC) in the context of surgery.

**Methods:**

DEX was given to lung cancer patients and its effects on expansion of monocytic MDSC (M-MDSC) were studied in the context of surgery. Spontaneous tumour metastasis was induced in C57BL/6 mice and the effects of DEX on M-MDSC expansion and metastasis formation were assessed.

**Results:**

DEX increased CD11b^+^CD33^+^HLA-DR^–^CD14^+^ M-MDSC in lung cancer patients after thoractomy. DEX-induced M-MDSC, in addition to have immunosuppressive activity, were more efficient in producing VEGF. Expansion of M-MDSC by DEX involved α_2_-adrenergic receptor. Using an experimental tumour metastasis mouse model, we demonstrated that the numbers of metastases on lung surface and CD11b^+^Ly6C^high^Ly6G^−^ M-MDSC during postoperative period were enhanced in DEX-treated mice. Promotion of tumour metastasis by DEX-induced M-MDSC involved VEGF, a key factor for tumour angiogenesis.

**Conclusions:**

DEX induces the proliferation of M-MDSC during postoperative period in lung cancer patients and this cell population is qualified with potent proangiogenic ability. Treatment of mice with DEX expands M-MDSC and promotes tumour metastasis through the increasing production of VEGF.

**Electronic supplementary material:**

The online version of this article (10.1186/s12967-018-1727-9) contains supplementary material, which is available to authorized users.

## Background

Dexmedetomidine (DEX) is a highly selective α_2_-adrenergic receptor (α_2_-AR) agonist with various effects on the central nervous system (CNS), including sedation, analgesia and anaesthetic-sparing effects [1]. Current indications have been expanded to include perioperative and procedural sedation [2]. Based on the effects of DEX on sympathetic and immunoregulatory, one can assume beneficial effects in the perioperative period of cancer patients. However, in vitro experiments have demonstrated that DEX promoted cancer cell survival and proliferation, and in vivo studies have showed promotive effect of DEX on tumour progression [3, 4]. Lavon et al. recently reported that DEX significantly promoted the tumour cell retention and the growth of metastases in rodent models in the context of surgery [5]. These findings call for mechanism studies to understand the deleterious effects of DEX.

Myeloid-derived suppressor cells (MDSC) are characterized by their myeloid origin, immature state, and most importantly by their potent ability to suppress immune responses, especially T cell proliferation and cytokine production [6]. MDSC consist of two subsets: granulocytic MDSC (G-MDSC) and monocytic MDSC (M-MDSC) [7]. G-MDSC are phenotypically and morphologically similar to neutrophils, whereas M-MDSC are similar to monocytes [8]. These cells represent a pathologic state of activation of monocytes and relatively immature neutrophils [9]. These cells are rare in steady-state conditions, but they accumulate abundantly during different pathologies and exert beneficial or deleterious effects to their progression. In recent years, MDSC are implicated in the promotion of tumour metastases by participating in the formation of premetastatic niches, promoting cell invasion and angiogenesis [10]. Our recent studies highlight an emerging role for MDSC in tumour metastasis that M-MDSC accumulate in lung cancer patients undergoing tumour resection and correlates with postoperative tumour metastasis [11, 12]. While the immunomodulating effects of DEX in cancer have been studied in depth, our understanding of their relevance for MDSC has not been assessed.

Since DEX is commonly used in intraoperative period of cancer patients, the facts that short-term perioperative interventions may affect long-term outcomes, together with causal findings from animal experiments, suggest that the perioperative period has a critical impact on cancer metastasis, and through it determines long-term outcomes [13]. This study aims to investigate the influences of the short-term use of clinically relevant doses of DEX on the progression of cancer metastases in the context of surgery and relevant mechanisms. Specifically, we hypothesized that DEX could promote metastasis through expanding M-MDSC during postoperative period.

## Subjects and methods

### Patients’ enrollment

A total of 103 adult lung cancer patients were prospectively enrolled at the First Affiliated Hospital of Kunming Medical University between July 2014 and July 2016, including 51 cases in the control group (Ctrl) and 52 cases in the dexmedetomidine (DEX) (Hengrui Medicine Co. Ltd, Nanjing, China) treatment group (see Table 1 for Patients’ characteristics). Patients received DEX pre- and intra-operatively with a median consumption of 122 µg (118–146 µg) using a microinfusion pump. Those who had palliative surgery or secondary malignancies were excluded from the analysis. Ethics Committee approval was obtained from the Internal Review Board of Kunming Medical University and a written informed consent was obtained in accordance with the declaration of Helsinki. Clinical data were collected from patient records and peripheral blood was drawn at preoperation (T_0_) and on postoperative day 1, 3 and 7 (depicted as T_1_, T_2_ and T_3_). Intraoperative anesthetic care of the patients comprised volatile-opioid general anesthesia.

**Table 1:** Patients’ characteristics

Clinicopathologic characteristics	Ctrl (n = 51)	DEX (n = 52)	*P* value
Age: median (minimum–maximum), years	59 (48–67)	63 (51–74)	0.615^a^
Gender: female/male	21/30	23/29	0.754^b^
Histology: adenocarcinoma/squamous cell carcinoma	38/13	44/8	0.203^b^
TNM stage: II/III	23/28	18/34	0.277^b^
Vascular invasion: present/absent	19/32	12/40	0.117^b^
Tumor size: < 3 cm/> 3 cm	25/26	30/22	0.378^b^
Tumor number: single/multiple	33/18	40/12	0.172^b^
Anesthesia duration: median (minimum–maximum), minutes	226 (168–422)	238 (184–454)	0.475^a^

^a^Mann–Whitney U test

^b^Pearson’s Chi square test

### Tumour cell line and mice

Mouse lewis lung carcinoma (LLC, H-2^b^) cells were cultured in RPMI 1640 medium (Gibco-BRL, Carlsbad, CA) supplemented with 10% fetal calf serum, 30 μg/mL gentamicin, and 0.2% sodium bicarbonate. Inbred female C57BL/6 mice (B6, H-2^b^) (8–10 weeks) were purchased from the Experimental Animal Institute of Peking Union Medical College.

### Flow cytometry analysis and cell sorting

To determine the frequency and phenotype of MDSC in peripheral blood mononuclear cells (PBMC) from lung cancer patients, flow cytometry (FCM) analysis was done using the following fluorescein-conjugated mouse anti-human monoclonal antibodies (mAb): FITC-CD11b (clone ICRF44), PE-Cy7-CD14 (clone M5E2), PE-CD33 (clone WM53) and PE-Cy5-HLA-DR (clone G46-6) (BD Pharmingen, San Diego, CA). Suspensions of lung cells from mice were prepared using an enzyme digestion method. Briefly, lungs of mice were perfused with 0.02% EDTA-PBS to wash blood vessels and incubated in RPMI 1640 medium containing collagenase/DNase I, and cell suspensions were washed. Lung cells and peripheral blood collected through tail vein were stained with different combinations of the following rat anti-mouse mAbs: FITC-CD11b (clone M1/70.15), PE-CD45 (clone 30-F11), PE-Cy5-Ly-6G (clone 1A8), PE-Cy7-Ly-6C (clone HK1.4) (BD Pharmingen). FCM was done on Beckman Coulter FC500 (San Jose, CA) and FCM data was analyzed using CXP software (Beckman Coulter). The percentage of CD11b^+^CD33^+^HLA-DR^−^CD14^−^ and CD11b^+^CD33^+^HLA-DR^−^CD14^+^ cells in PBMC of lung cancer patients was calculated according the formula: = % of CD11b^+^CD33^+^ cells × % of HLA-DR^−^CD14^–^ or HLA-DR^–^CD14^+^ cells in PBMC. FCM analysis of the expression of α_2_-AR on either human or mouse MDSC was performed using Biotin-conjugated mouse anti-human/mouse α_2_-AR mAb (clone S330A-51) (Novus Biologicals, Littleton, CO) and Streptavidin-BD Horizon™ PE-CF594 (BD Pharmingen).

Human PBMC were isolated from freshly heparinized peripheral blood from lung cancer patients by standard Ficoll density gradient centrifugation (Haoyang Biological Manufacture Co. Ltd, Tianjin, China). Isolation of CD11b^+^CD33^+^HLA-DR^−^, CD11b^+^CD33^+^HLA-DR^−^CD14^−^ and CD11b^+^CD33^+^HLA-DR^−^CD14^+^ cells was performed on FACSVantage SE cell sorter (Becton–Dickinson, Franklin Lakes, NJ). For cell sorting in mice, suspensions of lung cells were stained with fluorescein-conjugated rat anti-mouse CD11b, Ly6C and Ly6G mAb. Isolation of CD11b^+^Ly6C^low^Ly6G^+^ (G-MDSC) and CD11b^+^Ly6C^high^Ly6G^−^ (M-MDSC) was performed on FACSVantage SE cell sorter.

### Immunoassay for cytokines

The serum concentration of VEGF in lung cancer patients was detected with a commercial enzyme-linked immunosorbent assay (ELISA) kit (RayBiotech, Inc. Norcross, GA) according to the manufacturer’s instructions.

### MDSC suppression assay

The suppressive function of MDSC was assessed based on their ability to inhibit CD3-induced T cell activation. CD3^+^ cells were isolated from PBMC of lung cancer patients preoperatively using anti-CD3 magnetic beads (Miltenyi Biotec, Bergisch Gladbach, Germany) and plated at 2 × 10^5^ cells/well in 1 μg/mL of anti-CD3 Abs (muromonabCD3, Janssen Pharmaceutica, Titusville, NJ)-coated plates. Isolated CD11b^+^CD33^+^HLA-DR^−^, CD11b^+^CD33^+^HLA-DR^−^CD14^−^ and CD11b^+^CD33^+^HLA-DR^−^CD14^+^ cells (1 × 10^5^ cells/well) from the same lung cancer patient during perioperative period were added to the wells. Cell proliferation was determined 72 h later after incubating with ^3^H-thymidine for the last 16 h.

### Reverse transcription quantitative PCR

MDSC were isolated from lung cancer patients during perioperative period and total RNA was extracted using TaKaRa RNAiso Reagent (Takara Bio Inc. Otsu, Japan) according to the manufacturer’s instructions. A reverse transcription—polymerase chain reaction (RT-PCR) procedure was used to determine relative quantities of mRNA (One-step RT-PCR kit, Qiagen). The primers for all genes tested were synthesized by Invitrogen: VEGF 5′-CATTGGAGCCTTGCCTTG-3′ (sense) and 5′-TTCGTGGGGTTTCTGGTCT-3′ (antisense), GAPDH 5′-AGCCACATCGCTCAGACAC-3′ (sense) and 5′-GCCCAATACGACCAAATCC-3′ (antisense). For quantitative real-time PCR, cDNA (2 μL) reverse transcribed from total RNA was amplified by real-time PCR with 1 SYBR Green Universal PCR Mastermix (Bio-Rad). Each sample was analyzed in duplicate with the IQ-Cycler (Bio-Rad) and the normalized signal level was calculated based on the ratio to the respective GAPDH housekeeping signal.

To assess the relative mRNA expression of VEGF in mice, CD11b^+^Ly6C^low^Ly6G^+^ (G-MDSC) and CD11b^+^Ly6C^high^Ly6G^−^ (M-MDSC) cells were isolated from lungs of PBS (Ctrl), dexmedetomidine (DEX), or dexmedetomidine and yohimbine (DEX + YOH)-treated mice and total RNA was extracted using TaKaRa RNAiso Reagent according to the manufacturer’s instructions. A RT-PCR procedure was used to determine relative quantities of mRNA. The primers for all genes tested were synthesized by Invitrogen: VEGF 5′-GTACTTGCAGATGTGACAAGCCA-3′ (forward) and 5′-GGTGACATGGTTAATCGGTCTTT-3′ (reverse); GAPDH 5′-CCGGTGCTGAGTATGTCGT-3′ (forward) and 5′-CCTTTTGGCTCCACCCTTC-3′ (reverse). Each sample was analyzed in duplicate with the IQ-Cycler (Bio-Rad) and the normalized signal level was calculated based on the ratio to the respective GAPDH housekeeping signal.

### DEX tunes the differentiation of MDSC in vitro

CD11b^+^CD33^+^HLA-DR^−^ cells (3 × 10^4^ cells/well) isolated from peripheral blood of lung cancer patients 24 h after surgery using fluorescence-activated cell sorting (FACS) were cultured in RPMI 1640 medium, alone or cocultured with DEX (2.5 ng/mL), yohimbine (YOH, an α_2_-adrenergic antagonist) (2.5 ng/mL, see Additional file 1: Figure S1 for dose) (Sigma-Aldrich, St. Louis, MO), or DEX (2.5 ng/mL) and YOH (2.5 ng/mL) (DEX + YOH). A total of 24, 48 and 72 h after culture, floating cells were gently collected and numerated using a TC10 automated cell counter (Bio-Rad). The percentage of CD11b^+^CD33^+^HLA-DR^−^, CD11b^+^CD33^+^HLA-DR^−^CD14^−^ and CD11b^+^CD33^+^HLA-DR^−^CD14^+^ cells was analyzed by FCM and the absolute number of these cells was calculated according to the following formula: absolute number of CD11b^+^CD33^+^HLA-DR^−^, CD11b^+^CD33^+^HLA-DR^−^CD14^−^ and CD11b^+^CD33^+^HLA-DR^−^CD14^+^ cells = total number of cells harvested from each well × percent of CD11b^+^CD33^+^HLA-DR^−^, CD11b^+^CD33^+^HLA-DR^−^CD14^−^ and CD11b^+^CD33^+^HLA-DR^−^CD14^+^ cells (%).

### Experimental tumour metastasis model and surgical operation procedure

LLC spontaneous pulmonary metastasis experiments were performed as described previously [14]. Briefly, 1 × 10^6^ LLC cells in 0.1 mL of PBS were injected s.c. into the dorsa of C57BL/6 mice. When tumours were ~ 1500 mm^3^ in size, 14 days after LLC inoculation, the mice were randomly divided into three groups and treated with PBS (Ctrl group), dexmedetomidine (DEX group), or dexmedetomidine and yohimbine (2 mg/kg) (DEX + YOH group) [5]. Then the mice immediately underwent surgical removal of the tumor. To simulate the DEX clinical kinetics and effects, the dose 10 μg/kg h was chosen to reach an average plasma level of 1.5 ng/mL [5]. The DEX was administered using a slow release vehicle, which prevents initial high plasma levels and maintains prolonged exposure to the drugs. Mice received DEX pre- and intra-operatively with a median consumption of 0.5 µg (0.46–0.62 µg). All the mice in three groups were intraperitoneal injected with 0.8 mg/kg/mouse sodium pentobarbital (Sigma-Aldrich) for anesthesia. The day of tumor resection was designated as Day 0. Ten days after tumor resection, the mice were sacrificed, and their lungs were removed, and visible surface metastases were counted. The survival time of rest mice were continuously monitored for at least 60 days. All animal experiments were performed according to the guidelines and protocols approved by the Institutional Animal Care and Use Committee at Kunming Medical University.

### Immunohistochemistry

Tumour sections from mouse pulmonary metastatic nodules were incubated with rat anti-mouse CD31 mAb (clone MEC 13.3) (BD Pharmingen). A biotinylated rabbit anti-rat 2nd antibody was applied, followed by incubation with streptavidin-conjugated horseradish peroxidase (HRP). Peroxidase activity was localized with diaminobenzi dine (Shangon biotech Co. Ltd, Shanghai, China).

### Statistical analysis

GraphPad Prism 5.0 (La Jolla, CA) was used for data analysis. Results were presented as mean ± standard deviation (SD). Mann–Whitney U test and Pearson’s Chi square test were performed to evaluate the patient’s characteristics between Ctrl and DEX group. Unpaired two-tailed Student’s *t*-test was used to compare means between 2 groups. For comparison of individual time points, ANOVA was used for the comparisons among 3 or more groups. For the analysis of survival, log-rank Kaplan–Meier analysis was used. Differences were considered significant when *P* < 0.05.

## Results

### DEX expands M-MDSC in lung cancer patients after surgery

**Fig. 1:**
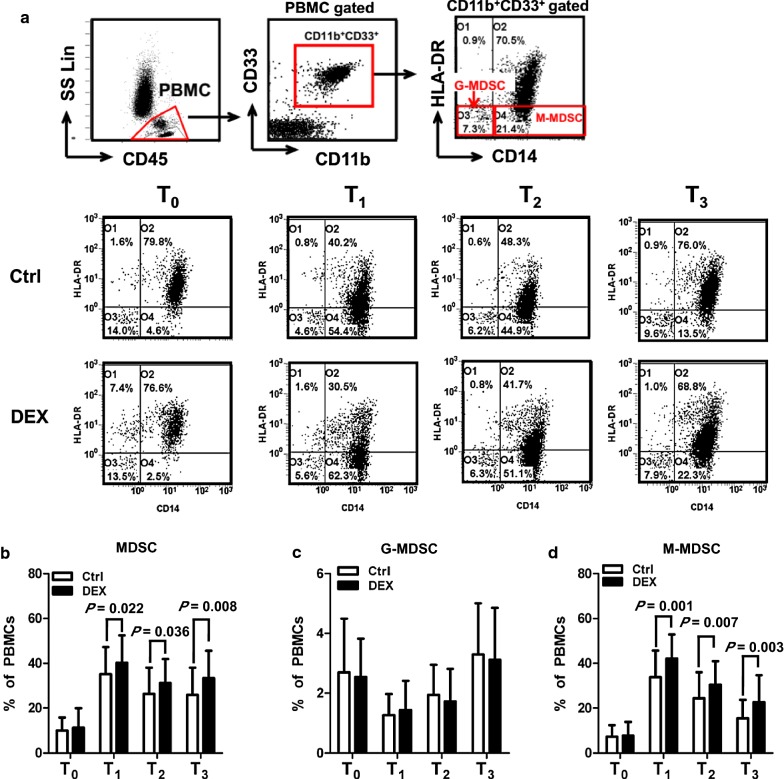
DEX expands M-MDSC after lung cancer surgery. A total of 103 lung cancer patients were divided into Ctrl (n = 51) and DEX (n = 52) group. The percentages of CD11b^+^CD33^+^HLA-DR^−^ (MDSC), CD11b^+^CD33^+^HLA-DR^−^CD14^−^ (G-MDSC) and CD11b^+^CD33^+^HLA-DR^−^CD14^+^ (M-MDSC) cells from peripheral blood mononuclear cells (PBMC) of lung cancer patients were analyzed by flow cytometry (FCM) at time of preoperatoin (T_0_), 1, 3 and 7 days after surgery (T_1_, T_2_ and T_3_). **a** Representative FCM data are shown. Frequency of (**b**) MDSC, **c** G-MDSC and **d** M-MDSC from PBMC of lung cancer patients during perioperative period

In human, MDSC are characterized by the cell surface expression of integrin CD11b, sialic acid binding lectin CD33 and low expression of HLA-DR [15]. Based on these surface markers, we found that while CD11b^+^CD33^+^HLA-DR^−^ cells (MDSC) circulate in lung cancer patients at baseline (preoperation, T_0_), the frequency of CD11b^+^CD33^+^HLA-DR^−^ cells was significantly increased on postoperative day 1, 3 and 7 (T_1_, T_2_ and T_3_, respectively) (Fig. 1a, b). When lung cancer patients were treated with DEX, we found that the percentage of CD11b^+^CD33^+^HLA-DR^−^ cells significantly increased as compared with patients in Ctrl group on postoperative days 1, 3 and 7 (Fig. 1b). CD11b^+^CD33^+^HLA-DR^−^ cells can be further divided into two subpopulations of cells expressing CD14^–^ or CD14^+^ (Fig. 1a). No significant differences in the percentage of CD14^–^ MDSC (G-MDSC) were found between Ctrl and DEX group during perioperative period (Fig. 1c). In contrast, the administration of DEX significantly increased the frequency of CD14^+^ MDSC (M-MDSC) on postoperative days 1, 3 and 7 (Fig. 1d). Thus, the administration of DEX promotes the expansion of M-MDSC in lung cancer patients.

### DEX-induced M-MDSC exert immunosuppressive and proagiogenic activity

**Fig. 2:**
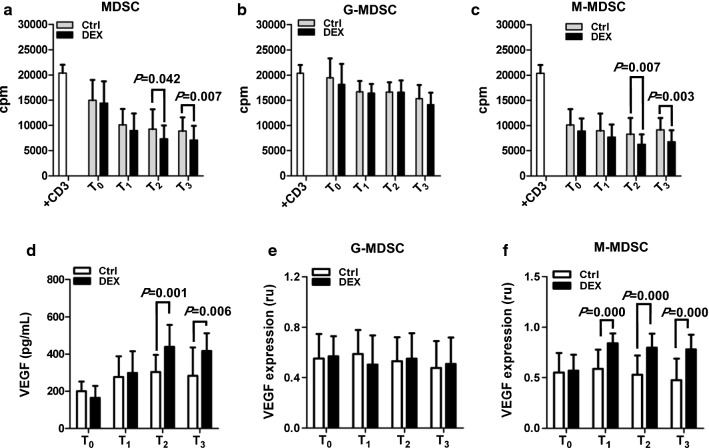
DEX-induced monocytic MDSC exerts immunosuppressive activity and produces VEGF. CD3^+^ cells were isolated from lung cancer patients (n = 16) preoperatively and plated at 2 × 10^5^ cells/well in 1 μg/mL of anti-CD3 Abs-coated plates in the presence of (**a**) CD11b^+^CD33^+^HLA-DR^−^ (MDSC), **b** CD11b^+^CD33^+^HLA-DR^−^CD14^+^ (M-MDSC) or **c** CD11b^+^CD33^+^HLA-DR^−^CD14^−^ (G-MDSC) cells (1 × 10^5^ cells/well) sorted from the same lung cancer patient at time of preoperatoin (T_0_), 1, 3 and 7 days after surgery (T_1_, T_2_ and T_3_). The proliferation of CD3^+^ cells was measured in triplicate by ^3^H-thymidine incorporation. **d** Serum concentration of VEGF in lung cancer patients of Ctrl (n = 16) and DEX (n = 16) group during perioperative period was measured with ELISA. **e** G-MDSC and **f** M-MDSC were isolated from lung cancer patients of Ctrl (n = 16) and DEX (n = 16) group during perioperative period and the relative mRNA expression of VEGF was analyzed by RT-qPCR. ru: relative units

We next evaluated the capacity of MDSC from DEX-treated patients to suppress T cell proliferation. In contrast with MDSC isolated from Ctrl group, MDSC in DEX group demonstrated higher level of T cell suppression (Fig. 2a). There was no difference in the T cell suppression of G-MDSC between Ctrl and DEX group during perioperative period (Fig. 2b). Although the M-MDSC isolated from either Ctrl or DEX group exerted immunosuppressive function against T cell proliferation, the M-MDSC in DEX-treated patients was more potent in inhibiting T cell proliferation on postoperative day 3 and 7 (Fig. 2c).

It has been reported that MDSC release VEGF and mediate tumour angiogenesis [11, 16]. In order to determine whether the administration of DEX was associated with higher level of VEGF, we first detected the levels of VEGF in the serum of Ctrl and DEX patients by ELISA. We found that the concentration of VEGF was elevated in serum from DEX-treated lung cancer patients as compared with Ctrl group on postoperative day 3 and 7 (Fig. 2d). The mRNA levels of VEGF were also measured in G-MDSC and M-MDSC. A similar pattern in mRNA expression level of VEGF was observed in G-MDSC isolated from patients of Ctrl and DEX group. In contrast, M-MDSC from DEX-treated patients had higher mRNA level of VEGF relative to Ctrl group (Fig. 2e, f).

### DEX expands M-MDSC by α_2_-AR receptor

**Fig. 3:**
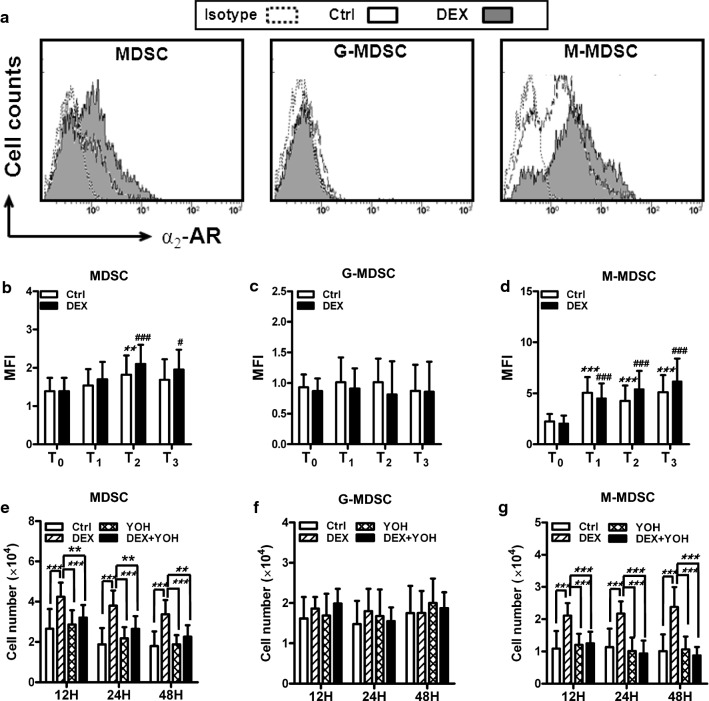
Expansion of M-MDSC by DEX is mediated via α_2_-AR. **a** Expression of α_2_-AR on CD11b^+^CD33^+^HLA-DR^−^ (MDSC), CD11b^+^CD33^+^HLA-DR^−^CD14^−^ (G-MDSC) and CD11b^+^CD33^+^HLA-DR^−^CD14^+^ (M-MDSC) was analyzed in peripheral blood mononuclear cells (PBMC) of lung cancer patients at time of preoperatoin (T_0_), 1, 3 and 7 days after surgery (T_1_, T_2_ and T_3_) by flow cytometry (FCM). Representative FCM data of α_2_-AR on MDSC, G-MDSC and M-MDSC on days 3 after tumor resection are shown. **b**–**d** Mean fluorescent intensity (MFI) of α_2_-AR on **b** MDSC, **c** G-MDSC and **d** M-MDSC was assessed. **P < 0.01 and ***P < 0.001 as compared with T_0_ in Ctrl group; ^#^*P* < 0.05 ^###^*P* < 0.001 as compared with T_0_ in DEX group. **e**–**g** CD11b^+^CD33^+^HLA-DR^−^ cells (3 × 10^4^ cells/well) isolated from lung cancer patients (n = 16) 24 h after surgery were cocultured with dexmedetomidine (DEX) (2.5 ng/mL), yohimbine (YOH, an α_2_-adrenergic antagonist) (2.5 ng/mL), or dexmedetomidine (2.5 ng/mL) and yohimbine (2.5 ng/mL) (DEX + YOH). Twelve, twenty-four and forty-eight hours after coculture, floating cells were gently collected and numerated using an automated cell counter. The percentage of MDSC, G-MDSC or M-MDSC was analyzed by FCM and the absolute number of these cells was calculated. *P < 0.05, **P < 0.01, ***P < 0.001

To investigate the mechanism behind DEX-mediated expansion of M-MDSC, we checked the expression of α_2_-AR, the cognate receptor for DEX, on MDSC. We found that MDSC constitutively expressed low level of α_2_-AR, and when lung cancer patients were undergone tumour resection, the expression of α_2_-AR on MDSC was elevated (Fig. 3a, b). After tumour resection and DEX administration, lung cancer patients had a significant increase of α_2_-AR on the MDSC and M-MDSC, but not G-MDSC (Fig. 3b–d). Moreover, the levels of α_2_-AR on the MDSC, G-MDSC and M-MDSC from Ctrl patients were similar to the levels seen in DEX-treated patients at the same time point (Fig. 3a–d).

To confirm the in vivo promotion of M-MDSC by DEX, we isolated CD11b^+^CD33^+^HLA-DR^−^ cells from lung cancer patients after surgery and performed a series of coculture experiments in vitro. We found that exogenous addition of DEX induced expansion of MDSC and M-MDSC, most consistently and significantly at 12 h after coculture (Fig. 3e–g). The addition of yohimbine (YOH, an α_2_-adrenergic antagonist) inhibited the expansion of MDSC and M-MDSC caused by DEX (Fig. 3e and g). Thus, DEX mediates the expansion of M-MDSC by binding to α_2_-AR.

### DEX induces M-MDSC and promotes tumour metastasis after surgery

**Fig. 4:**
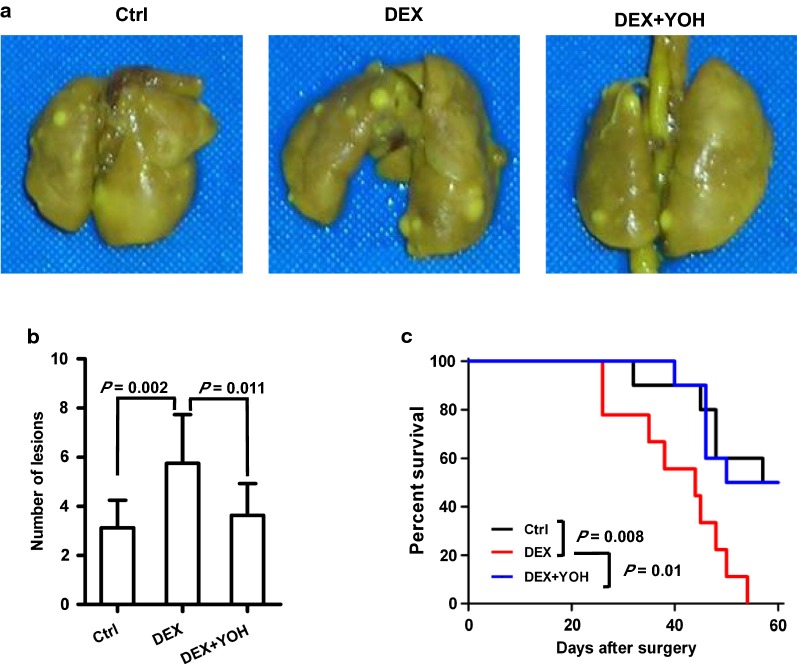
DEX promotes tumour metastases in mice exposed to tumour excision. 1 × 10^6^ LLC cells were injected s.c. into the dorsa of C57BL/6 mice. When tumours were ~ 1500 mm^3^ in size, 14 days after LLC inoculation, the mice were randomly divided into three groups and treated with PBS (Ctrl group), dexmedetomidine (DEX group), or dexmedetomidine and yohimbine (DEX + YOH group). Then the mice immediately underwent surgical removal of the tumor. All mice were sacrificed 10 days after surgery and the numbers of metastases on the lung surface were counted. **a** Typical examples of lung tissue. **b** The number of tumour lesions per lung (six mice per group). **c** Survival of mice in Ctrl, DEX and DEX + YOH group (ten mice per group). Data presented are representative of three replicated experiments

To assess the possible role of DEX in the prognosis of a tumour-bearing host undergoing tumour resection, C57BL/6 mice were treated with DEX. As showed in Fig. 4a, DEX administration systemically promoted the growth of metastatic lung tumours as quantified by counting metastatic nodules on pulmonary surface in the mice undergone tumour resection. Significant differences in the number of metastasis were found between Ctrl and DEX-treated mice 10 days after tumour resection (Fig. 4b). When DEX was given with YOH, a selective α_2_-AR antagonist, its effects on pulmonary metastasis were prevented (Fig. 4a, b). In a separate set of experiments, we evaluated whether the treatment of DEX had effects on the survival of mice undergone tumour resection. When the mice show dyspnea, they were euthanized and their lungs were removed. The freshly isolated lungs from Ctrl, DEX and “DEX + YOH” group had some replacement of the normal lung tissue by large metastatic nodules (Additional file 2: Figure S2A). All DEX-treated mice succumbed to the tumour metastasis within 2 months after the start of the tumour resection and DEX treatment. In contrast, 50% mice in Ctrl and “DEX + YOH” group were still alive at that time point (Fig. 4c). The lungs of survived mice from Ctrl and “DEX + YOH” group had less metastatic nodules (Additional file 2: Figure S2B).

**Fig. 5:**
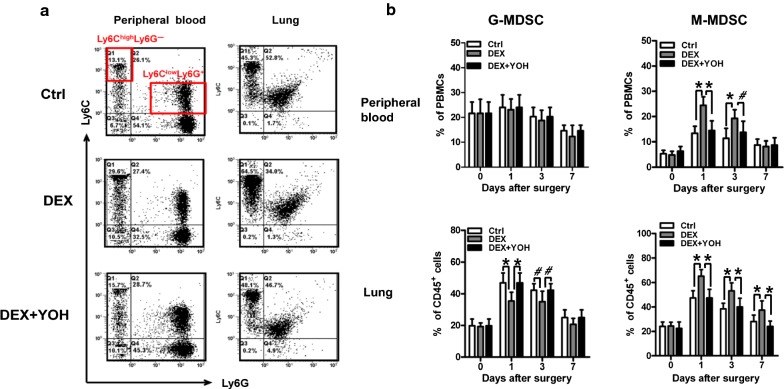
DEX expands M-MDSC in mice exposed to tumour excision. 1 × 10^6^ LLC cells were injected s.c. into the dorsa of C57BL/6 mice. When tumours were ~ 1500 mm^3^ in size, 14 days after LLC inoculation, the mice were randomly divided into three groups and treated with PBS (Ctrl), dexmedetomidine (DEX), or dexmedetomidine and yohimbine (DEX + YOH). Then the mice immediately underwent surgical removal of the tumor. The frequency of CD11b^+^Ly6C^low^Ly6G^+^ (G-MDSC) and CD11b^+^Ly6C^high^Ly6G^−^ (M-MDSC) cells in peripheral blood and lungs were analyzed on days 0, 1, 3 and 7 after surgery by flow cytometry (FCM). **a** Representative FCM data of G-MDSC and M-MDSC on day 1 after tumor resection are shown. **b** Frequency of G-MDSC and M-MDSC in peripheral blood and lungs were detected after tumour resection (three mice per group at each time point). Data presented are representative of three replicated experiments. ^#^*P* < 0.05, *P < 0.01

We then asked whether the induction and proliferation of M-MDSC could be affected following DEX administration. Mouse granulocytic and monocytic MDSC are defined as CD11b^+^Ly6C^low^Ly6G^+^ (G-MDSC) and CD11b^+^Ly6C^high^Ly6G^−^ (M-MDSC) (Fig. 5a) [8, 9]. Based on these cell surface markers, we found that after DEX treatment, mice had a significant increase of M-MDSC, but not G-MDSC, in their peripheral blood and lungs (Fig. 5b). The administration of YOH abrogated the expansion of CD11b^+^Ly6C^high^Ly6G^−^ cells in DEX-treated mice (Fig. 5a, b). Taken together, these results suggested that DEX expanded M-MDSC after surgical manipulation in tumour-bearing mice and promoted tumour metastasis. We also analyzed the expression of α_2_-AR on the mouse MDSC. There was a significant increase of α_2_-AR on the MDSC and M-MDSC of lungs of mice in Ctrl, DEX and “DEX + YOH” group after tumour resection (Additional file 3: Figure S3A, B). No significant differences in the expression of α_2_-AR on G-MDSC were found in Ctrl, DEX and “DEX + YOH” group during perioperative period (Additional file 3: Figure S3C).

### DEX induces VEGF in M-MDSC and promotes tumour angiogenesis

**Fig. 6:**
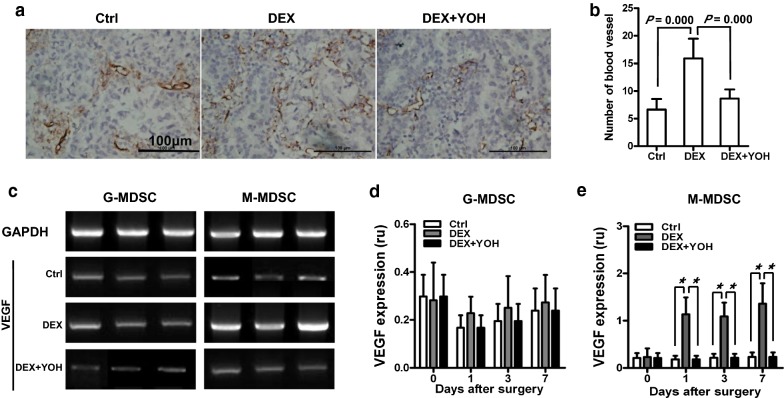
DEX mediates tumour angiogenesis via VEGF. **a** Immunohistochemistry staining of mouse pulmonary metastatic sections with CD31 antibody. Brown lines indicate tumour blood vessels. **b** Tumour blood vessels were counted in ten randomly selected fields (six mice per group). **c** Expression of VEGF in CD11b^+^Ly6C^low^Ly6G^+^ (G-MDSC) and CD11b^+^Ly6C^high^Ly6G^−^ (M-MDSC) cells isolated from lungs of mice was analyzed by RT-qPCR. Representative results of RT-qPCR from G-MDSC and M-MDSC isolated from lungs of mice on day 1 after surgery were shown. **d**, **e** G-MDSC and M-MDSC were isolated from lungs of mice on days 0, 1, 3 and 7 after surgery and the relative mRNA expression of VEGF was analyzed by RT-qPCR (three mice per group at each point). Data presented are representative of three replicated experiments. *P < 0.001. ru, relative units

To investigate whether increased vascularity could explain the increased tumour metastssis in the DEX-treated mice, we stained tumour samples with anti-CD31 antibody. Significant higher blood vessel density was observed in metastatic nodules derived from DEX-treated mice after surgery than those from Ctrl and “DEX + YOH”-treated mice, with an average of (6.63 ± 1.80), (15.88 ± 3.37) and (8.63 ± 1.58) respectively (Fig. 6a, b). To evaluate the proagiogeneic activity of G-MDSC and M-MDSC, we examined the mRNA levels of VEGF in Ctrl, DEX- and “DEX + YOH”-treated mice (Fig. 6c). No significant differences in the mRNA expression of VEGF in the G-MDSC were found between Ctrl, DEX- and “DEX + YOH”-treated mice during perioperative period (Fig. 6d). However, CD11b^+^Ly6C^high^Ly6G^−^ cells isolated from lungs of mice treated with DEX produced higher mRNA level of VEGF as compared with Ctrl and “DEX + YOH”-treated mice (Fig. 6e).

## Discussion

This study focused on the effect of DEX on cancer metastasis in the context of surgery, similar to the perioperative setting of DEX used in cancer patients [17]. We found that after lung cancer surgery the peripheral blood of DEX-treated patients was of higher accumulation of monocytic MDSC and consequently these cells were more efficient in suppressing T cell proliferation and producing proangiogenic factor VEGF. These modulation effects were dependent on the expression of α_2_-AR on M-MDSC. We also investigated the influence of surgical resection on the generation of M-MDSC and the growth of tumour metastasis using an experimental mouse tumour model, and demonstrated that the numbers of metastases on lung surface and M-MDSC during postoperative period were increased in DEX-treated mice. Taken together, these data suggest that DEX-induced M-MDSC during postoperative period were qualified with potent proangiogenic and tumour promotive activity.

Following a surgery, cellular immunity remains suppressed for several days with decrease in circulating levels of cytotoxic T lymphocytes, dendritic cells, natural killer cells and helper T cells (Th) [13, 18, 19]. The magnitude of this immune suppression is proportional to the degree of surgical manipulation [20]. Recent experimental and clinical evidences suggest that DEX has been associated with reduced inflammatory cytokine release, modulation of inflammatory transcription factors, oxidative stress and inflammatory cells [21]. A randomized controlled trial in gastric cancer patients who had gastrectomies indicates that DEX, given intraoperatively, has potent immunomodulatory properties that are observed as improvement in the Th1/Th2 ratio and reduction in IL-6 and tumor necrosis factor (TNF) [22]. Similar results were reported in other randomized controlled trials that DEX alleviates the reduction of cellular immunity, thereby ameliorating the impaired immune functions in patients who had mastectomy or radical surgery of colon carcinoma [23–25]. In addition to cell-mediated immunity, the immunosuppressive components in peripheral and premetastatic tissue have been showed potent tumour-promoting activity [26]. Therefore, fully disclosing the mechanisms responsible for mediating the effects of surgical stress on tumour mass is crucial for determining the full effect of DEX administration on tumour metastasis and for devising effective interventions. We report that, increasing level of CD11b^+^CD33^+^HLA-DR^−^ myeloid cells was observed in DEX-treated patients and meanwhile, these cells exhibited inhibition against T cell proliferation. Further characterization of the MDSC subsets that are present in lung cancer patients revealed that, while CD14^+^ expressing M-MDSC derived from both Ctrl and DEX-treated patients are increased, the in vitro suppressive function of M-MDSC was more potent in DEX-treated patients. Although a considerable amount of data support that DEX alleviates the inhibition of cellular immunity [27], and this immunoregulatory function are also found in tumours [28], DEX appears to exert an immunosuppressive effect in the setting of surgery. Therefore, the net effect of immunoregulatory of DEX during perioperative period could be a key factor may contribute substantially to the risk of subsequent emergence of tumour relapse and metastasis.

Another characteristic of tumor metastasis is that the formation of premetastatic niche correlates with immunosuppressive cells accumulation and subsequently formation of new blood vessels [29]. Among these immunosuppressive cells, myeloid cells such as MDSC have been proved to be a potent promoter in proangiogenesis and tumour metastasis [30]. MDSC isolated from mouse tumours displayed activated Stat3 and induced angiogenesis in an in vitro tube formation assay via Stat3 induction of angiogenic factors, including VEGF [31]. In the context of surgery, CD11b^+^CD33^+^HLA-DR^−^-expressing MDSC significantly increased in lung cancer patients after thoracotomy and MDSC isolated after surgery from lung cancer patients were more efficient in promoting angiogenesis via VEGF [11]. Thus, DEX-induced MDSC may also induce formation of blood vessels and therefore promote tumour metastasis after the primary tumour removing. Our finding that high numbers of M-MDSC expanded by DEX produces high levels of VEGF sheds some mechanistic light on this issue.

The immunomodulatory properties of DEX could be beneficial in the context of inflammatory conditions that require sedation, such as sepsis, ischemia–reperfusion injury and ventilator-associated lung injury, among many others [1, 2]. Nonetheless, in contrast to these findings, some researchers have noted that the immunomodulatory effects of DEX can be beneficial for the growth of tumours [3, 4, 32]. Takefumi et al. report that DEX inhibits antitumour immunity as shown by the accelerated tumour growth, possibly through the decreased production of IL-12 from antigen presenting cells, resulting in a Th2 shift and decreased cytotoxic T lymphocytes (CTL) activity [33]. Cata et al. retrospectively analyzed data from 1404 operated patients with non-small cell lung cancer (NSCLC), of which 241 were treated with DEX perioperatively [34, 35]. The use of DEX was associated with statistically and clinically significant lower survival rates at 5 year postsurgery, an effect evident only in patients receiving DEX. A consensus result was observed in the present study that DEX increased the growth of metastases of a mouse lung carcinoma, and this resulted in a shorter survival time in DEX-treated mice. Whether DEX definitively enhances or inhibits tumour growth is still to be resolved, since differences in the experimental design, cancer histologic cell type, and DEX administration schedule used can all affect the experimental outcome. In clinical setting, cancer patients are exposed to DEX during surgery for removal of the primary tumour, and/or for up to a day following it [17]. Thus, the potential effects of DEX on long-term cancer outcomes would most likely be mediated through its indirect effects on a residual disease, especially on preexisting micro-metastases in the metastatic prone tissue/organ. To test whether the short-term effects of DEX on M-MDSC expansion translate to long-term effects, we applied a mouse model quantifying metastases following tumour resection. DEX increased the metastasis number in the spontaneous pulmonary experiment, indicating the biological significance of a single exposure to DEX in the context of micrometastases, and supporting the hypothesis that the effects of a relatively short perioperative exposure to DEX could have detrimental effects on long-term clinical cancer prognosis. The drug schedules of DEX employed were chosen to simulate clinical plasma levels, and behavioural and physiological effects.

Previous studies have shown that a number of immune cells functions have been altered by α_2_-AR, but there has been no report regarding the involvement of α_2_-AR in the expansion of MDSC. α_2_-Adrenoceptors are present in T lymphocytes, and agonists to this receptor decrease lymphocyte proliferation and both interferon-γ and IL-4 production [36]. DEX could reduce IL-2 production in macrophages and led to a decreased ratio of helper T lymphocytes subsets, Th1 to Th2 [33]. Natural killer cells also show increased cytotoxic activity in response to α_2_-AR agonists [37]. We may also deduce the mechanism of DEX with the expansion of M-MDSC in surgery context, since it is established that DEX can modulate the JAK/STAT signaling pathway which may be the final transcription factor involved in the expansion of M-MDSC [38–42]. In stressed circumstances, the effects of DEX were initiated quickly following its administration, and attenuated along with cessation of its behavioural effects, suggesting immediately inducible and reversible mechanisms. Thus, immune mechanisms might include reduced antimetastatic immunity and simultaneously induced prometastatic immunity that can quickly and transiently be modulated, which controls tumor metastases.

A better understanding of immune regulatory functions of DEX, which include the mechanisms by which DEX induces immunosuppressive ingredients and promotes tumour metastasis, has a potential to lead to new avenues for preventive intervention in cancer surgery by reducing their immunosuppressive effects and preventing tumour metastasis. Most nonsteroidal anti-inflammatory drugs (NSAIDs) function as cyclooxygenase-2 (COX-2) inhibitors that inhibit production of prostaglandin E2 (PGE2) [43]. Because PGE2 induces expansion of MDSC, Fujita et al. reported that in mouse glioma model, treatment with the COX-2 inhibitors celecoxib or acetylsalicylic acid (ASA) suppress gliomagenesis by inhibiting MDSC development and accumulation in the tumor microenvironment [44]. Moreover, when applied to tumor-bearing hosts or added to MDSC cultured in a tumor microenvironment, the NSAID indomethacin (IND) induced MDSC to differentiate into a cell population with reduced suppressive activity, which favored the development of a more efficient antitumor response [45]. The possibility to modulate MDSC using NSAIDs offers an opportunity for treating a wide variety of pathologic conditions, especially cancer, without the need of depleting these cells from the host.

## Conclusions

In the context of surgery, the systemic administration of DEX effectively induces the proliferation of monocytic MDSC which possess proangiogenic and immunosuppressive activity and may promote tumour metastasis. This suggests that DEX administration may exert a deleterious effect for the prognosis of lung cancer patients via modulation of premetastatic niche. Prospective randomized controlled trials are needed to properly assess the effects of DEX on lung cancer patients and other cancers, and animal studies should further elucidate potential mediating mechanisms to be tested in cancer patients.

## Additional files

**Additional file 1: Figure S1.** Expansion of M-MDSC by dexmedetomidine is inhibited by yohimbine. (A, B) CD11b^+^CD33^+^HLA-DR^−^ cells (3 × 10^4^ cells/well) isolated from lung cancer patients (n = 6) 24 h after surgery were cocultured with **(A)** dexmedetomidine (DEX) or **(B)** dexmedetomidine and yohimbine (DEX + YOH). Twenty-four hours after coculture, floating cells were collected and numerated using an automated cell counter. The percentage of CD11b^+^CD33^+^HLA-DR^−^CD14^+^ (M-MDSC) was analyzed by flow cytometry and the absolute number of M-MDSC was calculated.

**Additional file 2: Figure S2.** DEX promotes tumour metastases in mice exposed to tumour excision. 1 × 10^6^ LLC cells were injected s.c. into the dorsa of C57BL/6 mice. When tumors were 1500 mm^3^ in size, the mice were divided into 3 groups and treated with PBS (Ctrl group), dexmedetomidine (DEX group), or dexmedetomidine and yohimbine (DEX + YOH group). Then the mice immediately underwent surgical removal of the tumor. **(A)** When the mice showed dyspnea, they were euthanized and their lungs were removed. **(B)** On day 60 after tumor resection, the survived mice from Ctrl and DEX + YOH group were euthanized and their lungs were removed.

**Additional file 3: Figure S3.** Expression of α_2_-AR on mouse MDSC. LLC cells were injected s.c. into the dorsa of C57BL/6 mice. When tumours were 1500 mm^3^ in size, the mice were divided into 3 groups and treated with PBS (Ctrl), DEX or DEX and YOH (DEX + YOH). Then the mice immediately underwent surgical removal of the tumor. Expression of α_2_-AR was analyzed on days 0, 1, 3 and 7 (depicted as T_0_, T_1_, T_2_ and T_3_) after surgery on the CD11b^+^Gr-1^+^ (MDSC), CD11b^+^Ly6C^high^Ly6G^−^cells (M-MDSC) and CD11b^+^Ly6C^low^Ly6G^+^ (G-MDSC) of lungs of mice (three mice per group at each time point) by flow cytometry. Mean fluorescent intensity (MFI) of α_2_-AR on **(A)** MDSC, **(B)** M-MDSC and **(C)** G-MDSC was assessed. *P < 0.001 as compared with T_0_ in Ctrl group; ^#^*P* < 0.001 as compared with T_0_ in DEX group; ^†^*P* < 0.001 as compared with T_0_ in DEX + YOH group.

## Abbreviations

α_2_-AR: α_2_-adrenergic receptor
DEX: dexmedetomidine
ELISA: enzyme-linked immunosorbent assay
FACS: fluorescence-activated cell sorting
FCM: flow cytometry
IL: interleukin
MDSC: myeloid-derived suppressor cells
MFI: mean fluorescence intensity
PBMC: peripheral blood mononuclear cells
PBS: phosphate-buffered saline
RT-qPCR: reverse-transcription quantitative polymerase chain reaction
VEGF: vascular epithelial growth factor
YOH: yohimbine
