# Supplementary material for: Dexmedetomidine expands monocytic myeloid-derived suppressor cells and promotes tumour metastasis after lung cancer surgery
Source: J Transl Med. 2018 Dec 11;16:347. doi: 10.1186/s12967-018-1727-9 (PMC6288950; doi:10.1186/s12967-018-1727-9)
Supplement: Supplementary file 1 — Additional file 1: Figure S1. Expansion of M-MDSC by dexmedetomidine is inhibited by yohimbine. (A, B) CD11b+CD33+HLA-DR− cells (3 × 10^4^ cells/well) isolated from lung cancer patients (n = 6) 24 h after surgery were cocultured with (A) dexmedetomidine (DEX) or (B) dexmedetomidine and yohimbine (DEX + YOH). Twenty-four hours after coculture, floating cells were collected and numerated using an automated cell counter. The percentage of CD11b+CD33+HLA-DR−CD14+ (M-MDSC) was analyzed by flow cytometry and the absolute number of M-MDSC was calculated. [file 12967_2018_1727_MOESM1_ESM.pdf]

Additional file 1: Figure S1

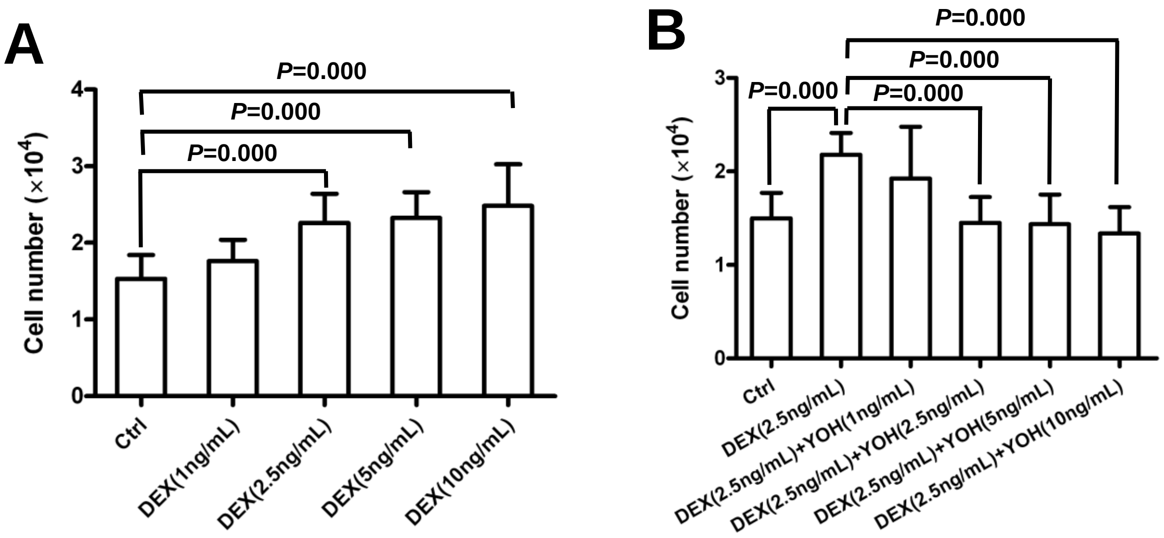

Additional file 1: Figure S1. Expansion of M-MDSC by dexmedetomidine is inhibited by yohimbine. (A-B) $CD11b^+CD33^+HLA\text{-}DR^-$ cells (3×10^4 cells/well) isolated from lung cancer patients (n = 6) 24 hours after surgery were cocultured with **(A)** dexmedetomidine (DEX) or **(B)** dexmedetomidine and yohimbine (DEX+YOH). Twenty-four hours after coculture, floating cells were collected and numerated using an automated cell counter. The percentage of $CD11b^+CD33^+HLA\text{-}DR^-CD14^+$ (M-MDSC) was analyzed by flow cytometry and the absolute number of M-MDSC was calculated.
