# Supplementary material for: Dexmedetomidine expands monocytic myeloid-derived suppressor cells and promotes tumour metastasis after lung cancer surgery
Source: J Transl Med. 2018 Dec 11;16:347. doi: 10.1186/s12967-018-1727-9 (PMC6288950; doi:10.1186/s12967-018-1727-9)
Supplement: Supplementary file 2 — Additional file 2: Figure S2. DEX promotes tumour metastases in mice exposed to tumour excision. 1 × 10^6^ LLC cells were injected s.c. into the dorsa of C57BL/6 mice. When tumors were 1500 mm3 in size, the mice were divided into 3 groups and treated with PBS (Ctrl group), dexmedetomidine (DEX group), or dexmedetomidine and yohimbine (DEX + YOH group). Then the mice immediately underwent surgical removal of the tumor. (A) When the mice showed dyspnea, they were euthanized and their lungs were removed. (B) On day 60 after tumor resection, the survived mice from Ctrl and DEX + YOH group were euthanized and their lungs were removed. [file 12967_2018_1727_MOESM2_ESM.pdf]

Additional file 2: Figure S2

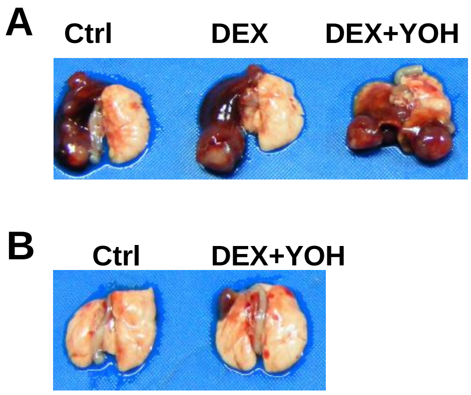

Additional file 2: Figure S2. DEX promotes tumour metastases in mice exposed to tumour excision. 1×10^6 LLC cells were injected s.c. into the dorsa of C57BL/6 mice. When tumors were 1,500 mm^3 in size, the mice were divided into 3 groups and treated with PBS (Ctrl group), dexmedetomidine (DEX group), or dexmedetomidine and yohimbine (DEX+YOH group). Then the mice immediately underwent surgical removal of the tumor. **A.** When the mice showed dyspnea, they were euthanized and their lungs were removed. **B.** On day 60 after tumor resection, the survived mice from Ctrl and DEX+YOH group were euthanized and their lungs were removed.
