# Supplementary material for: Dexmedetomidine expands monocytic myeloid-derived suppressor cells and promotes tumour metastasis after lung cancer surgery
Source: J Transl Med. 2018 Dec 11;16:347. doi: 10.1186/s12967-018-1727-9 (PMC6288950; doi:10.1186/s12967-018-1727-9)
Supplement: Supplementary file 3 — Additional file 3: Figure S3. Expression of α2-AR on mouse MDSC. LLC cells were injected s.c. into the dorsa of C57BL/6 mice. When tumours were 1500 mm3 in size, the mice were divided into 3 groups and treated with PBS (Ctrl), DEX or DEX and YOH (DEX + YOH). Then the mice immediately underwent surgical removal of the tumor. Expression of α2-AR was analyzed on days 0, 1, 3 and 7 (depicted as T0, T1, T2 and T3) after surgery on the CD11b+Gr-1+ (MDSC), CD11b+Ly6ChighLy6G−cells (M-MDSC) and CD11b+Ly6ClowLy6G+ (G-MDSC) of lungs of mice (three mice per group at each time point) by flow cytometry. Mean fluorescent intensity (MFI) of α2-AR on (A) MDSC, (B) M-MDSC and (C) G-MDSC was assessed. *P < 0.001 as compared with T0 in Ctrl group; #P < 0.001 as compared with T0 in DEX group; †P < 0.001 as compared with T0 in DEX + YOH group. [file 12967_2018_1727_MOESM3_ESM.pdf]

Additional file 3: Figure S3

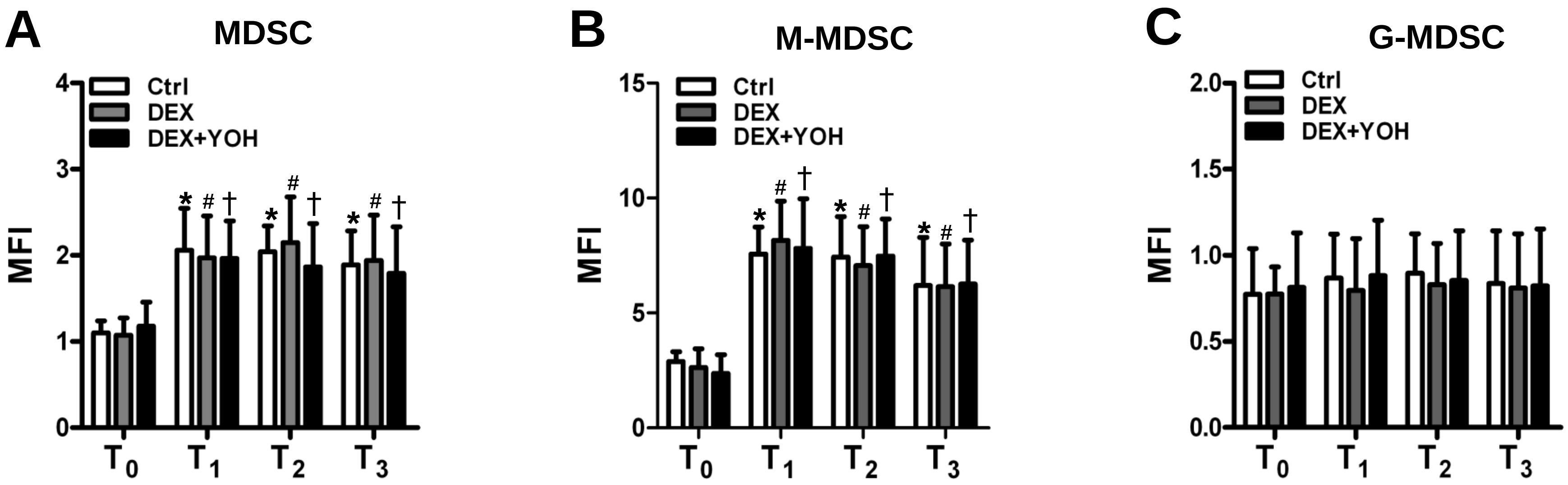

Additional file 3: Figure S3. Expression of α_2-AR on mouse MDSC. LLC cells were injected s.c. into the dorsa of C57BL/6 mice. When tumours were 1,500 mm^3 in size, the mice were divided into 3 groups and treated with PBS (Ctrl), DEX or DEX and YOH (DEX + YOH). Then the mice immediately underwent surgical removal of the tumor. Expression of α_2-AR was analyzed on days 0, 1, 3 and 7 (depicted as T_0, T_1, T_2 and T_3) after surgery on the $CD11b^+Gr\text{-}1^+$ (MDSC), $CD11b^+Ly6C^{high}Ly6G^{-}$cells (M-MDSC) and $CD11b^+Ly6C^{low}Ly6G^+$ (G-MDSC) of lungs of mice (three mice per group at each time point) by flow cytometry. Mean fluorescent intensity (MFI) of α_2-AR on **(A)** MDSC, **(B)** M-MDSC and **(C)** G-MDSC was assessed. *$P < 0.001$ as compared with T_0 in Ctrl group; # $P < 0.001$ as compared with T_0 in DEX group; **†** $P < 0.001$ as compared with T_0 in DEX+YOH group.
